# Systematic reviews of observational studies of Risk of Thrombosis and Bleeding in General and Gynecologic Surgery (ROTBIGGS): introduction and methodology

**DOI:** 10.1186/s13643-021-01814-2

**Published:** 2021-10-08

**Authors:** Lauri I. Lavikainen, Gordon H. Guyatt, Yung Lee, Rachel J. Couban, Anna L. Luomaranta, Ville J. Sallinen, Ilkka E. J. Kalliala, Paul J. Karanicolas, Rufus Cartwright, Riikka L. Aaltonen, Kaisa Ahopelto, Karoliina M. Aro, Ines Beilmann-Lehtonen, Marco H. Blanker, Jovita L. Cárdenas, Samantha Craigie, Päivi J. Galambosi, Herney A. Garcia-Perdomo, Fang Zhou Ge, Huda A. Gomaa, Linglong Huang, Matthew L. Izett-Kay, Kirsi M. Joronen, Päivi K. Karjalainen, Nadina Khamani, Tuomas P. Kilpeläinen, Antti J. Kivelä, Tapio Korhonen, Hanna Lampela, Anne K. Mattila, Borna Tadayon Najafabadi, Taina P. Nykänen, Carolina Nystén, Sanna M. Oksjoki, Sanjay Pandanaboyana, Negar Pourjamal, Chathura B. B. Ratnayake, Aleksi R. Raudasoja, Tino Singh, Riikka M. Tähtinen, Robin W. M. Vernooij, Yuting Wang, Yingqi Xiao, Liang Yao, Jari Haukka, Kari A. O. Tikkinen

**Affiliations:** 1 Faculty of Medicine, University of Helsinki, Helsinki, Finland; 2 Department of Health Research Methods, Evidence and Impact, McMaster University, Hamilton, ON Canada; 3 Department of Medicine, McMaster University, Hamilton, ON Canada; 4 Division of General Surgery, Department of Surgery, McMaster University, Hamilton, ON Canada; 5 Department of Anesthesia, McMaster University, Hamilton, ON Canada; 6 Department of Obstetrics and Gynecology, University of Helsinki and Helsinki University Hospital, Helsinki, Finland; 7 Department of Transplantation and Liver Surgery, University of Helsinki and Helsinki University Hospital, Helsinki, Finland; 8 Department of Gastroenterological Surgery, University of Helsinki and Helsinki University Hospital, Helsinki, Finland; 9 Department of Metabolism, Digestion and Reproduction, Imperial College London, London, UK; 10 Department of Surgery, Sunnybrook Health Sciences Centre, Toronto, ON Canada; 11 Department of Surgery, University of Toronto, Toronto, ON Canada; 12 Department of Obstetrics and Gynaecology, LNWH NHS Trust, London, UK; 13 Department of Obstetrics and Gynecology, Turku University Hospital and University of Turku, Turku, Finland; 14 Department of General Practice and Elderly Care Medicine, University Medical Center Groningen, University of Groningen, Groningen, The Netherlands; 15 National Center for Health Technology Excellence (CENETEC) Direction of Health Technologies assessment, Mexico City, Mexico; 16 Division of Urology, Department of Surgery, School of Medicine, Universidad del Valle, Cali, Colombia; 17 Michael G. DeGroote School of Medicine, McMaster University, Hamilton, ON L8S 4L8 Canada; 18 High Institute of Public Health, Alexandria University, Alexandria, Egypt; 19 Tanta Chest Hospital, Ministry of Health and Population, Tanta, Egypt; 20 Urogynaecology Department, The John Radcliffe Hospital, Oxford University Hospitals, Oxford, UK; 21 Department of Obstetrics and Gynecology, Central Finland Central Hospital, Jyväskylä, Finland; 22 Faculty of Health Sciences, University of Eastern Finland, Kuopio, Finland; 23 Department of Obstetrics and Gynecology, Institute of Childrens’ Health, I.M. Sechenov First Moscow State Medical University (Sechenov University), Moscow, Russia; 24 Department of Urology, University of Helsinki and Helsinki University Hospital, Helsinki, Finland; 25 Experts by Experience, Abdominal Center, Helsinki University Hospital, Helsinki, Finland; 26 Central Finland Central Hospital, Department of Surgery, Jyväskylä, Finland; 27 Department of Surgery, Hyvinkää Hospital, Hyvinkää, Finland; 28 Felicitas Mehiläinen Turku, Turku, Finland; 29 Department of HPB and Transplant Surgery, Freeman Hospital, Newcastle Upon Tyne, UK; 30 Population Health Sciences Institute, Newcastle University, Newcastle Upon Tyne, UK; 31 Laboratory of Molecular Oncology, Faculty of Medicine, University of Helsinki, Helsinki, Finland; 32 Department of Surgery, The University of Auckland, Auckland, New Zealand; 33 Auckland City Hospital, Auckland, New Zealand; 34 Department of Obstetrics and Gynecology, Tampere University Hospital, Tampere, Finland; 35 Julius Center for Health Sciences and Primary Care, University Medical Center Utrecht, Utrecht University, Utrecht, The Netherlands; 36 Department of Nephrology & Hypertension, University Medical Center Utrecht, Utrecht, The Netherlands; 37 West China School of Nursing/Department of Nursing, West China Hospital, Sichuan University, Chengdu, China; 38 Faculty of Medicine and Health Technology, Tampere University, Tampere, Finland; 39 Clinicum/Department of Public Health, University of Helsinki, Helsinki, Finland; 40 Department of Surgery, South Karelian Central Hospital, Lappeenranta, Finland

**Keywords:** Baseline risk, Bleeding, Gynecology, Hemorrhage, Modeling, Risk of bias, Surgery, Surgical complications, Thromboprophylaxis, Thrombosis

## Abstract

**Background:**

Venous thromboembolism (VTE) and bleeding are serious and potentially fatal complications of surgical procedures. Pharmacological thromboprophylaxis decreases the risk of VTE but increases the risk of major post-operative bleeding. The decision to use pharmacologic prophylaxis therefore represents a trade-off that critically depends on the incidence of VTE and bleeding in the absence of prophylaxis. These baseline risks vary widely between procedures, but their magnitude is uncertain. Systematic reviews addressing baseline risks are scarce, needed, and require innovations in methodology. Indeed, systematic summaries of these baseline risk estimates exist neither in general nor gynecologic surgery. We will fill this knowledge gap by performing a series of systematic reviews and meta-analyses of the procedure-specific and patient risk factor stratified risk estimates in general and gynecologic surgeries.

**Methods:**

We will perform comprehensive literature searches for observational studies in general and gynecologic surgery reporting symptomatic VTE or bleeding estimates. Pairs of methodologically trained reviewers will independently assess the studies for eligibility, evaluate the risk of bias by using an instrument developed for this review, and extract data. We will perform meta-analyses and modeling studies to adjust the reported risk estimates for the use of thromboprophylaxis and length of follow up. We will derive the estimates of risk from the median estimates of studies rated at the lowest risk of bias. The primary outcomes are the risk estimates of symptomatic VTE and major bleeding at 4 weeks post-operatively for each procedure stratified by patient risk factors. We will apply the Grading of Recommendations Assessment, Development and Evaluation (GRADE) approach to rate evidence certainty.

**Discussion:**

This series of systematic reviews, modeling studies, and meta-analyses will inform clinicians and patients regarding the trade-off between VTE prevention and bleeding in general and gynecologic surgeries. Our work advances the standards in systematic reviews of surgical complications, including assessment of risk of bias, criteria for arriving at the best estimates of risk (including modeling of the timing of events and dealing with suboptimal data reporting), dealing with subgroups at higher and lower risk of bias, and use of the GRADE approach.

**Systematic review registration:**

PROSPERO CRD42021234119

**Supplementary Information:**

The online version contains supplementary material available at 10.1186/s13643-021-01814-2.

## Background

### Introduction

Surgeons perform over 300 million operations annually worldwide [1]. Venous thromboembolism (VTE) encompasses two related conditions: deep venous thrombosis (DVT) and pulmonary embolism (PE). These represent severe and sometimes fatal complications of surgery [2]. VTE has earlier been described as the leading cause of preventable hospital-related death [3, 4]. It has been suggested that the mortality burden of VTE could be as high as 500,000 deaths in Europe and 100,000 to 300,000 deaths in the USA every year [5]. In contrast, some studies have suggested a substantial overdiagnosis and overtreatment of VTE [6, 7]. Post-operative bleeding can lead to transfusion and/or reoperation, and is associated with a higher risk of death and complications as well as longer hospital stay and increased health care costs [8, 9].

An earlier systematic review and meta-analysis that pooled general, urologic, and gynecologic surgery patients suggested that pharmacological prophylaxis decreases the risk of VTE by approximately 50%, while increasing the risk of major post-operative bleeding by a similar percentage [10–12]. The decision to use thromboprophylaxis represents, therefore, a trade-off between the decreased risk of VTE and the increased risk of bleeding.

This trade-off depends crucially on the risk of VTE and major bleeding when the patient is not receiving anticoagulation (we refer these risks baseline risks) [13]. When the baseline risk for VTE is high and the risk for bleeding is low, a 50% reduction in VTE represents a substantial benefit. The impact of prophylaxis will, however, result in net harm for patients with low risk of VTE and high risk of bleeding. In urology, the baseline risks for VTE and bleeding vary according to procedure-specific factors [10–12]. The risks also vary between different general and gynecologic procedures [14, 15]; the magnitude of this variation is, however, uncertain. This makes the decision regarding the use of thromboprophylaxis difficult.

Table 1 presents major guidelines addressing the use of thromboprophylaxis for abdominal and/or pelvic surgery [2, 10, 16–34]. Thromboprophylaxis guidelines in general (abdominal) or gynecologic surgery have not generally made procedure-specific recommendations (Table 1). There are three exceptions in abdominal and pelvic surgery, two of them in general or gynecologic surgery: (i) American Society of Hematology (ASH) 2019 guidelines gave procedure-specific recommendation for laparoscopic cholecystectomy [2], and (ii) Enhanced Recovery After Surgery Society (ERAS) gave procedure- (but not approach-) specific recommendations for pancreaticoduodenectomy [29, 30]. The only completely procedure- (and approach-) specific guideline in abdominal and/or pelvic surgery is the European Association of Urology (EAU) guidelines, providing procedure-specific thromboprophylaxis recommendations for urologic surgery procedures . Overall, experts have failed to achieve a consensus regarding the use of VTE prophylaxis in general and gynecologic surgery [2, 10, 16–34]. The significant practice variation within and between countries is therefore unsurprising [35–37].

**Table 1:** Major guidelines on the use of thromboprophylaxis for abdominal and/or pelvic surgery

Guideline association or guideline group	Year	Type of surgery	Stratification by procedure (Yes/No)	Number (percentage) of procedure specific recommendations for abdominal and/or pelvic surgery^**a**^	Number (percentage) of procedure specific recommendations for all surgeries^**a**^
Enhanced Recovery After Surgery Society (ERAS)	2020	Vulvar and vaginal	No	0	0
American Society of Clinical Oncology (ASCO)	2019	Major cancer	No	0	0
American Society of Hematology (ASH)	2019	All	Partly^b^	3 (60%)	5 (38%)
International Initiative on Thrombosis and Cancer (ITAC-CME)	2019	Cancer	No	0	0
Enhanced Recovery After Surgery (ERAS) Society	2018	Elective colorectal	No	0	0
National Institute for Health and Care Excellence (NICE) (of the United Kingdom)	2018	All	No^c^	0	14 (30%)
Southern African Society of Thrombosis and Hemostasis	2018	Obstetrics and gynecology	No	0	0
The American Society of Colon and Rectal Surgeons	2018	Colorectal	No	0	0
Asian Venous Thrombosis Forum (AVTF) working group	2017	All	No	0	0
European Association of Urology (EAU)	2017	Urology	Yes	23 (100%)	23 (100%)
European Society of Anesthesiology (ESA)	2017	All	No^d^	0	6 (21%)
Enhanced Recovery After Surgery Society (ERAS)	2016	Gynecologic oncology	No	0	0
Enhanced Recovery After Surgery Society (ERAS)	2016	Liver surgery	No	0	0
Thrombosis Canada	2016	Non-orthopedic	No	0	0
The Scottish Intercollegiate Guidelines Network (SIGN)	2014	General abdominal Gynecologic Bariatric	No^e^	0	6 (21%)
Enhanced Recovery After Surgery Society (ERAS)	2013	Pancreaticoduodenectomy	Yes^f^	1 (100%)	1 (100%)
Enhanced Recovery After Surgery Society (ERAS)	2013	Radical cystectomy	Yes^f^	1 (100%)	1 (100%)
Enhanced Recovery After Surgery Society (ERAS)	2013	Elective rectal/pelvic	No	0	0
American College of Chest Physicians (ACCP)	2012	Non-orthopedic	No	0	0
National Health and Medical Research Council (NHMRC) (of Australia)	2012	All	No^g^	0	10 (34%)
American Urological Association (AUA)	2009 (reviewed 2011)	Urologic	No	0	0
American College of Obstetricians and Gynecologists (ACOG)	2007	Gynecologic	No	0	0

Selected guidelines provide guidance for thromboprophylaxis in abdominal and/or pelvic surgery. Table presents number of procedure-specific recommendations regarding decision to give or not to give pharmacological or mechanical prophylaxis in each guideline

^a^ Of the prioritized clinical questions regarding pharmacological/mechanical prophylaxis vs. no pharmacological/mechanical prophylaxis

^b^ Five procedure-specific recommendations for laparoscopic cholecystectomy, transurethral resection of the prostate, radical prostatectomy (approach not specified), total hip arthroplasty and total knee arthroplasty

^c^ Fourteen procedure-specific recommendations for elective hip replacement, elective knee replacement, non-arthroplasty orthopedic knee surgery, lower limb amputation, and varicose vein surgery

^d^ Six procedure-specific recommendations for cesarean section, total hip arthroplasty, total knee arthroplasty, coronary artery bypass graft, bioprosthetic aortic valve implantation surgery, and abdominal aortic aneurysm repair

^e^ Six procedure-specific recommendations for total hip replacement, total knee replacement, coronary artery bypass graft, and varicose vein surgery

^f^ Approach not specified

^g^ Ten procedure-specific recommendations for cesarean section, total hip replacement, total knee replacement, and knee arthroscopy

Before initiating this review, our research group has completed series of systematic reviews in urology that informed the first ever patient risk and procedure-specific guideline, the European Association of Urology (EAU) Guideline on Thromboprophylaxis in Urological Surgery [11, 12, 38]. Therefore, the fact that general and gynecologic surgical thromboprophylaxis guidelines are not procedure-specific (Table 1) is unsurprising. The key reason for this lack of procedure-specific guidance is the absence of high-quality systematic reviews providing procedure-specific estimates of VTE and bleeding risk. We aim to fill this knowledge gap by performing a series of systematic reviews, modeling studies and meta-analyses informing clinicians and patients about the trade-off between VTE prevention and bleeding in general and gynecologic surgeries. We call this initiative the Risk Of Thrombosis and Bleeding In General and Gynecologic Surgery (ROTBIGGS). Each review will summarize the evidence of the procedure-specific frequencies of VTE and major bleeding in general (abdominal) and gynecologic surgeries. Our work will also advance the methods of systematic reviews of surgical complications. In this article, we describe the specific methods used in our upcoming systematic reviews.

## Methods/design

Our study protocol was registered in the International Prospective Register of Systematic Reviews (PROSPERO CRD42021234119). We have adhered to the Preferred Reporting Items for Systematic Reviews and Meta-Analysis Protocols (PRISMA-P) guidance [39] to report this protocol (Additional file 1).

### Eligibility criteria

We will include retrospective and prospective observational studies and case series enrolling adult patients undergoing a target procedure in general or gynecologic surgery but exclude randomized trials. Observational studies of unselected patients are likely the best sources of estimates of VTE and bleeding [40, 41]. We will not include randomized controlled trials (RCTs); although RCTs provide the best estimates of treatment efficacy, they do not provide best estimates about the baseline risks of patients in routine practice. Although randomization minimizes the bias in allocating the patients to the intervention and control groups, there are other biases inherent to RCTs that limit their generalizability. Inclusion and exclusion criteria of the trials often lead to highly selected patient populations, and RCTs may therefore be limited when estimating baseline risks. For instance, patients with advanced age and greater comorbidity as well as those from lower socioeconomic status are under-represented in RCTs [42, 43].

Expert panels have already selected the most relevant procedures for this study (Table 2, see additional files for classification and related terms used). The expert panelists were experienced consultant surgeons from Canada, Finland, and the UK representing general, colorectal, HPB, and upper-GI surgery (V.J.S., P.J.K., K.A., I.B-L., A.J.K., H.L., A.K.M., T.P.N., S.P.); cancer and non-cancer gynecology (ALL, IEJK, RC, RLA, KMA, JLC, PJG, MLI-K, KMJ, PKK, SMO, RMT); and (clinician-)methodologists (LIL, GHG, KAOT). We will include studies with patients undergoing at least one of these procedures (Table 2). We will further stratify these procedures by approach (such as open, laparoscopic or robotic) and other criteria, if we find enough studies providing these estimates. As we did not include obstetric surgery, we will exclude peripartum procedures or surgeries performed due to obstetric reasons. We will exclude studies in which over 10% of participants underwent any other procedure than the procedure of interest, when the outcomes of interest are not stratified between procedures/approaches.

**Table 2:** General and gynecologic surgery procedures included in the search

General surgery	Gynecology
1. Colectomy	1. Vulvectomy (hemivulvectomy)
2. Proctocolectomy	2. Trachelectomy (cervicectomy)
3. Abdominoperineal resection	3. Cervical conization
4. Hernia repair (groin, umbilical)	4. Adnexal surgery
5. Hernia repair (ventral)	5. Sterilization
6. Cholecystectomy	6. Hysterectomy
7. Gastrectomy	7. Surgery for ovarian cancer
8. Pancreaticoduodenectomy	8. Pelvic exenteration
9. Distal pancreatectomy	9. Surgery for vaginal cancer (colpectomy)
10. Liver resection	10. Myomectomy
11. Appendectomy	11. Colposuspension
12. Sleeve gastrectomy	12. Sacrocolpopexy
13. Gastric bypass	13. Transvaginal mesh
14. Splenectomy (elective)	14. Vaginal pelvic organ prolapse surgery (without mesh)
15. Small bowel resection	15. Perineoplasty
16. Rectopexy	16. Mid-urethral tape/sling
	17. Urethral bulking
	18. Deep endometriosis surgery
	19. Uterine artery embolization
	20. Hysteroscopy
	21. Dilatation and curettage
	22. Transvaginal oocyte retrieval

We will only include studies with at least 50 adult patients per target procedure. Small studies tend to show different, often greater, treatment effects than larger studies [44]. There are many possible reasons for these discrepancies such as publication bias, selective reporting of results and higher risk of bias [45, 46].

We will include studies that report at least one absolute risk estimate of our outcomes: fatal PE, symptomatic non-fatal PE, symptomatic DVT, symptomatic VTE, major bleeding, or fatal bleeding. Absolute estimates include the percentages (e.g., 2.0%), proportions (e.g., 0.01), natural units or natural frequency (e.g., 2 in 1000 patients), and natural frequency per time (e.g., 4 in 1000 patient years).

Studies must also be generalizable to the population of interest (that is, adult patients undergoing a target procedure). To assess the applicability and representativeness of each study, and the heterogeneity between the estimates, we will record the mean age of the study population and the proportion of patients with malignant disease. We will exclude studies with atypical populations for either of these factors when clear outliers exist. We will also exclude studies with high risk of bias when enough studies with low risk of bias for a target procedure are identified for a target procedure (“Final selection of eligible studies (risk of bias and outliers)” section). Finally, in case of overlapping study populations, we will primarily include the article with lowest risk of bias and secondarily the article with greater number of patients.

### Outcomes

The primary outcomes will be the procedure-specific absolute risks of symptomatic VTE and major bleeding. While conducting our earlier systematic reviews on procedure-specific risks of thrombosis and bleeding in urology, we recognized substantial heterogeneity in the definitions of major bleeding across different studies [11, 12, 38]. Based on the bleeding features independently associated with 30-day mortality [47] and iterative discussion, we will use three different major bleeding definitions: (1) bleeding requiring reoperation (including exploration and angioembolization), (2) bleeding leading to the transfusion of one or more units of red blood cells, and (3) bleeding leading to post-operative hemoglobin below 70 g/L. In addition, we will measure the absolute risks of fatal pulmonary embolism and fatal bleeding.

We will use cumulative risk estimates at post-operative day 28. We chose this 4-week time frame for estimates of risks of thrombosis and bleeding because this is a feasible and frequently selected time frame for prophylaxis [48, 49]. When the authors present the frequency of events at more than one time point, we will record the number of events up to three months and use the absolute risk closest to 4 weeks. All outcomes will be extracted and analyzed separately for each procedure. Surrogate outcomes such as asymptomatic DVT and the amount of estimated blood loss during the operation are of questionable importance to patients [50], and we will not collect data on such events.

### Study selection and data abstraction

#### Literature search, study selection, application of eligibility criteria, and data abstraction

We will perform comprehensive searches separately for general and gynecologic surgeries with the aid of an information specialist (R.J.C.). A combination of keyword and medical subject headings (MeSH) search will include the post-operative complications and VTE block combined with the surgery block (including the target surgery subfield terms combined with the target procedure and body part terms).

Our searches will include Embase, MEDLINE, Web of Science, and Google Scholar from January 1, 2004, until the present for general surgery and from January 1, 2000 until the present for gynecologic surgery. We limit our searches to contemporary studies, because the baseline risks of VTE and bleeding have likely changed over time [51–53]. The update search is planned for 1 year before estimated publication date of results articles.

We will also search for any reviews (found by our search) that may include observational studies of our interest. We will review these for possible eligible studies and add identified studies to selection process. We will further identify articles for procedures by reviewing the reference lists of the included studies. Finally, we will include articles that were not yielded by the search but are known to the experts in the panel to the full text screening.

**Fig. 1:**
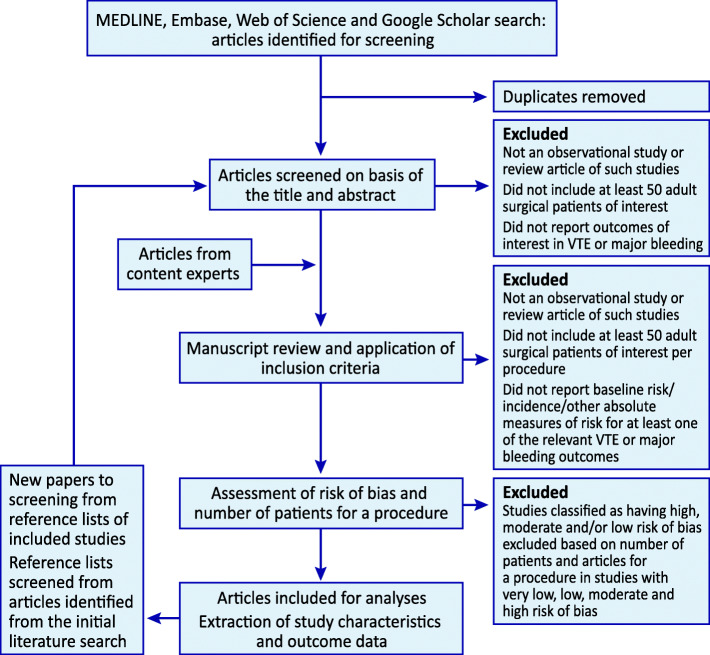
Study flow chart

We developed piloted, standardized data forms for screening articles and data extraction. Methodologically trained reviewers will apply these forms, guided with written instructions, to screen study reports for eligibility and extract data from eligible reports. The reviewers will conduct pilot screening and data extraction exercises to achieve a high level of agreement. Reviewers will screen abstracts in DistillerSR (Evidence Partners, Ottawa, Canada), an online systematic review software program. Reviewers will first screen titles and abstracts to select papers for full-text assessment and then full-text papers to confirm the eligibility of the articles (Fig. 1). All screening and data extraction will be completed independently and in duplicate. In every full-text screening pair, there will be at least one clinician expert (general/abdominal surgeon in general surgery and gynecologic surgeon in gynecologic surgery). An adjudicator (lead author or clinician-methodologist) will resolve any disagreement on eligibility or data extraction.

Because of the very large number of studies, our data extraction will be conducted in two phases. In the first phase, we will assess the risk of bias and classify studies as having very low, low, moderate or high risk of bias (“Assessment of risk of bias and outliers (final selection of eligible studies)” section). In this first phase, we will also extract data regarding procedure characteristics: procedure name, number of patients and outcomes reported for each procedure. In the second phase, (i) if we have at least 1000 patients and five articles for a target procedure in studies with very low and low risk of bias, we will exclude studies with moderate and high risk of bias; (ii) if we have at least 2000 patients and 10 articles for a certain target procedure in studies with very low, low, or moderate risk of bias, we will exclude studies with high risk of bias; and (iii) in other situations, we will use all studies irrespective of their risk of bias. These thresholds will be specific for approach of the target procedure. Finally, we will conduct these aforementioned steps separately for each outcomes of interest.

For all eligible studies, we will collect information on several other characteristics of the studies and their populations that may be predictive of VTE or major bleeding (Table 3). Finally, we will send our consensus of data extraction to the original authors of each article for confirmation or correction. When necessary, we will ask the authors to clarify details regarding the thromboprophylaxis, surgical technique, and other missing or unclear information.

**Table 3:** Characteristics assessed

Characteristics	
Age	Use of anticoagulants^a^
Gender	Use of aspirin or other antiplatelets^b^
Proportion of patients with malignancy	Use of mechanical thromboprophylaxis^c^

^a^ Including warfarin, low molecular weight heparin, low dose unfractionated heparin, dabigatran, apixaban, betrixaban, edoxaban, rivaroxaban, fondaparinux, danaparoid and lepirudin

^b^ Including aspirin, clopidogrel, prasugrel, ticlopidine, dipyridamole, ticagrelor, cilostazol, tirofiban, vorapaxar as well as thromboxane inhibitors, thromboxane synthase inhibitors, thromboxane receptor antagonists, and terutroban

^c^ Including antithrombosis stockings, intermittent pneumatic compression devices, and foot-pumps

#### Assessment of risk of bias and outliers (final selection of eligible studies)

We will assess the methodological features that could potentially bias the estimates of VTE or bleeding risk. Criteria for risk of bias are still poorly established for studies on baseline risk compared to studies on therapeutic interventions [54–57]. Risk of bias criteria are also dependent on the outcomes of interest and other factors specific to this study. Through iterative discussion and consensus-building and informed by the prior literature [11, 12, 38, 58–60], we developed an instrument for the risk of bias assessment to allow classifying studies as having very low, low, moderate, or high risk of bias (Table 4). The criteria for the overall risk of bias are based on the number of individual risk of bias domains that are judged as having high risk of bias (Table 4).

**Table 4:** Design features used for assessment of risk of bias

Domain^**a**^	Low risk of bias	High risk of bias
**Sampling**	Consecutive patient recruitment or administrative database with random sampling	Non-consecutive patient recruitment or administrative database with non-random sampling
**Thromboprophylaxis documentation**	Reporting of patients’ thromboprophylaxis	No reporting of patients’ thromboprophylaxis
**Source of information**	Prospective data collection by study investigators Retrospective duplicate chart reviews with good documentation of agreement between reviewers	Retrospective duplicate chart reviews without documentation of agreement between reviewers Administrative database information
**Recruitment years**	Studies with the majority of patient recruitment years 2010 or after	Studies with the majority of patient recruitment years 2009 or before
**Specification of length of follow-up**	Studies that clearly define the time period of follow-up (up to 3 months)	Studies that do not clearly define the time period of follow-up
**Study type**	International multicenter; Multicenter in one country; Single center, not single surgeon	Single surgeon series
**Overall risk of bias**^**a**^	No high risk of bias domains: Very low risk of bias One high risk of bias domain: Low risk of bias Two high risk of bias domains: Moderate risk of bias Three or more high risk of bias domains: High risk of bias	

^a^We will use the overall risk of bias as eligibility criteria when there are a sufficient number of patients in studies with low risk of bias for a given procedure

Because the baseline risks of VTE and bleeding are likely to have changed over time [61–63], older studies present potential risk of bias when estimating the present baseline risks. The complication estimates depend on the length of follow-up and studies that do not clearly define the time period of follow-up may therefore be biased. In addition, the details of thromboprophylaxis are sometimes insufficiently reported in available studies, which may also bias the results. We will estimate the thromboprophylaxis used based on information from the authors and, if not directly provided, on data on the type of thromboprophylaxis typically used in the country during the study period. Since the exclusion criteria include atypical and selected populations, we will not assess risk of bias resulting from the exclusion of patients.

### Analysis

#### Choosing the best estimate

To assess the baseline risks of VTE and major bleeding, we will use the median values of estimates from the eligible studies. The medians were chosen over pooled estimates across studies, because the pooled estimates would give more weight to larger studies. However, very large studies are also likely to have factors idiosyncratic to that population and setting, influencing the risks of both VTE and bleeding. Very large study may have poor methodological quality and dominate the smaller studies at less risk of bias. Thus, there is little reason to allow the large studies to have substantially more weight than the small ones. Heterogeneity in a systematic review of observational studies is greater than in a systematic review of randomized trials and, thus, included studies may not be similar enough to justify pooling [64]. Under these circumstances, the median is likely to provide better estimates of typical risk than is the pooled estimate [65].

#### Modeling the risk of VTE over time

We will use cumulative risk estimates at post-operative day 28 for our procedure-stratified estimates for the risks of VTE and major bleeding. For the studies that do not report VTE estimates using this interval, we will use a similar method described in our earlier systematic reviews to adjust the absolute VTE risk by post-operative day [11, 12, 38].

To improve the trustworthiness of our VTE risk over time model, we will conduct a separate systematic review and update this (VTE risk over time) model based on the best available evidence regarding the risk and time course of VTE by post-operative day. We will include prospective studies that report at least 20 symptomatic, post-operative VTE (or DVT/PE) events for at least two time points within 90 days post-surgery. We will include studies in which at least 95% of patients probably underwent a surgical procedure in one or more of the following fields/specialties: orthopedics, general/gastrointestinal surgery, thoracic surgery, urologic surgery, gynecologic surgery (excluding obstetrics), plastic surgery, hand surgery, breast surgery, endocrine surgery, and/or transplant surgery. We will include only studies that recruited patients in the year 2000 or later.

In this VTE risk over time model, primary outcome will be the occurrence of VTE on each of the 28 days (4 weeks) following surgery. Secondary outcomes include (i) occurrence of VTE on each of the 90 days (3 months) following surgery, (ii) occurrence of DVT on each day of the 28 days following surgery, and (iii) occurrence of PE on each day of the 28 days following surgery. We will record the timing of VTE by day as reported in each individual study. We will then model the timing of VTE for each individual study using natural cubic spline interpolation and R data analysis language. We will pool these models to a single model using the random effects inverse variance method. In our main model, we will adjust for the thromboprophylaxis use. In addition, we will perform a secondary analysis without any adjustments as well as sensitivity analyses in which we will revise the reported results in each study modeling what would have happened under various conditions of thromboprophylaxis. We will perform three such sensitivity analyses by assuming (i) 1 week of thromboprophylaxis for all patients, (ii) 2 weeks of thromboprophylaxis for all patients, and (iii) 3 weeks of thromboprophylaxis for all patients. If the study reports thromboprophylaxis, we will include the study only in the sensitivity analysis in which the practice corresponds most closely to the sensitivity analysis assumption.

#### Modeling the risk of bleeding over time

For the studies that do not report their bleeding estimates at 4 weeks, we will use the model previously described in our earlier systematic reviews to adjust the absolute bleeding risk by post-operative day [11, 12, 38]. This bleeding risk over time model shows that 90% of the 30-day bleeding events happen during the first week. Therefore, for studies providing bleeding estimates at time period of 31 to 90 days, we will assume constant risk of bleeding beyond the first post-operative week.

#### Calculating risks

To produce up-to-date information, we will extract data from contemporary observational studies. However, the contemporary studies representing the current rates of VTE often include patients that receive thromboprophylaxis. In addition, the decreasing incidence of VTE, linked to improving surgical techniques, adopting early mobilization, and extended prophylaxis as the standard of care [51–53], represents a challenge in estimating the efficacy of thromboprophylaxis.

To adjust the reported risks of VTE and bleeding for the use of thromboprophylaxis, we will estimate the effectiveness of prophylaxis. To achieve this, we will update our previously conducted meta-analyses of placebo-controlled randomized trials on the relative risks of VTE and bleeding among those who receive prophylaxis [11, 12, 38]. We will estimate the effects of antithrombotic agents such as unfractionated heparin, low molecular weight heparin, direct oral anticoagulants, and aspirin as well as mechanical prophylaxis (Table 3).

We will then adjust the reported risk by multiplying the relative risk by the reported risk in the proportion of patients who received prophylaxis. For instance, if a study including only patients that receive thromboprophylaxis for 4 weeks (time point of our primary outcome) shows a VTE risk of 1%, assuming that the relative risk reduction with prophylaxis is approximately 50% (as stated earlier, we will update the systematic review on this issue and relative effect estimates may change), we will infer that the included patients would have experienced a 2% risk of VTE had they not received prophylaxis.

Similarly, if the same study shows the bleeding risk of 3%, and because anticoagulant prophylaxis increases this risk by approximately 50%, we will infer the bleeding risk of 2% without prophylaxis. If thromboprophylaxis is used for less than 4 weeks, we will estimate its impact using same rationale but will also consider the duration of thromboprophylaxis by using our bleeding risk over time model [11, 12, 38].

If the study does not provide estimates of VTE but only DVT, PE, or both, we will calculate the risk of VTE by using the following approach described in our earlier systematic reviews [11, 12, 38]. We will review data from studies that reported DVT, PE, and VTE totals and estimate the overlap (i.e., patients with both DVT and PE) from these studies. We will then apply the degree of overlap to estimate VTE frequency in studies that provided only separate reports of DVT, PE, or both.

#### Stratifying the risks of VTE and bleeding according to procedure risk factors

We will stratify the risks of VTE for each procedure (see Table 2), further stratifying these procedures according to approach used (open, laparoscopic, robotic) and other procedure specific factors (e.g., subtotal vs. total; minor vs. major; or elective vs. emergency). This stratification will depend on how the included studies stratify the procedures and on recommendations from methodologists and expert consultant surgeons.

#### Stratifying the risk of VTE and bleeding according to patient risk factors

After assessing the procedure-specific baseline risk of VTE, we will stratify the risk by patient-related risk factors. We will use the tool described in our earlier systematic reviews [11, 12, 38]. We will assess four risk factors: (1) age 75 or more, (2) obesity (body mass index of 35 or more), (3) VTE in a first degree relative (parents, full siblings, or children)—all of these increase the risk approximately two-fold—and (4) prior VTE, with risk ratio of approximately 4. Patients with any combination of two or more risk factors will be assumed to have a risk ratio of 4. Using these risk factors, we will then categorize risk of VTE as low, medium (risk ratio of 2), and high risk (risk ratio of 4).

To calculate the estimates of absolute risks for these groups, for each procedure we will estimate the proportion of patients having each of the risk factors using eligible studies. To estimate the proportion of patients aged 75 or more, we will first estimate the mean/median age and SD for a target procedure, and then assume a normal distribution and calculate the proportion of patients aged 75 or more. To estimate the proportion of patients with body mass index 35 or more, we will first estimate the mean/median age and SD for a target procedure, and then use population-based data on distribution of body mass index and calculate the proportion of and patients with body mass index 35 or more. We will calculate the proportion of those with a personal history of VTE by using population-based study providing cumulative risks of personal history of VTE by age. Finally, we will estimate the proportion of patients with family history of VTE by using data from Michigan Surgical Quality Collaborative study [66]. Our earlier search did not reveal studies demonstrating convincing and replicable risk factors for bleeding [38]. We will therefore not stratify bleeding risk by patient-specific factors.

#### Case fatality

We will also estimate case fatality rates for VTE and major bleeding. For the case fatality of VTE, we will divide the number of fatal PE by the number of symptomatic VTE using studies that provide both estimates. For the case fatality of major bleeding, we will divide the number of fatal bleeding by the number of major bleeding.

#### Certainty of estimates

We will use the Grading of Recommendations Assessment, Development, and Evaluation (GRADE) approach to rate the certainty of evidence (also referred to as “quality of evidence” and “confidence in estimates”) [67–69]. The GRADE system classifies the certainty of evidence in one of four levels—high, moderate, low, and very low. For treatment effect, RCTs begin as high-quality evidence and observational studies as low-quality evidence. The certainty of evidence from observational studies addressing a question of prognosis (such as risk of VTE or bleeding post-surgery) begins as high quality but may be rated down for several reasons [57, 70]. We will assess and rate down for (i) risk of bias, (ii) inconsistency of results, (iii) indirectness of evidence, and (iv) imprecision, ending up rating overall certainty in estimates as very low, low, moderate, or high. We will also assess uncertainties in our study design and rate down the certainty of evidence accordingly: in all cases, we will rate down to moderate owing to potential uncertainties in our VTE and bleeding risk over time models and model of patient risk strata [12]. We will not rate down for publication bias as it is very difficult both to determine the magnitude of such bias, and to place a threshold for its likely presence in observational research [71].

### Patient involvement

Two patient partners (T.K. and C.N.; one who had undergone a general surgery and one a gynecologic surgery procedure) participated in the designing of the project, especially in the selection of outcome measures. They will also participate in the designing of the upcoming plain language summaries and leaflets for dissemination of results to the public. Both are co-authors of this protocol article.

## Discussion

Our work will use rigorous methods to make the best use of available observational data. We have specified explicit eligibility criteria, conducted comprehensive searches, and created an instrument for risk of bias assessment specific for this review. Teams of two reviewers will independently assess eligibility, risk of bias, and extract data. At least one clinician expert will assess the eligibility for every article in the full text screening phase. The lead author or a clinician-methodologist will adjudicate any discrepancies and we will send our consensus of data extraction to the original authors of each article for confirmation or correction. We will carefully address the many challenges involved in generating baseline risks of VTE and bleeding from low-quality observational data. We will conduct meta-analyses to adjust for the use of thromboprophylaxis and differences in the length of follow up. We will also take into account patient risk factors of VTE and bleeding outcomes. We will exclude studies that would bias our results and, at the same time, find balance for the amount of data for estimates. Our research group has earlier performed this kind of endeavor in urologic surgery [11, 12]. This work informed the first ever procedure-specific thromboprophylaxis guideline in any field of surgery, endorsed by the European Association of Urology . Our current project will explore this very frequent clinical dilemma in two large-scale surgical specialties: general abdominal and gynecologic surgery. We will implement improvements to the prior endeavor methods, including conducting more comprehensive literature searches and updating the VTE risk over time model.

There will be limitations to our work. As our reviews will reflect the published literature, we are unable to exclude the possibility of publication bias completely. Second, there is uncertainty in our models to get estimates for baseline risks of VTE and bleeding. Third, the large number of patients in contemporary practice receiving some form of thromboprophylaxis, and the paucity of studies that used the same follow-up duration mandate use of modeling with its inherent limitations. Fourth, indexing of observational research is inferior to that of randomized trials, leaving possible that our search missed some relevant studies. Fifth, surgical studies often focus on disease-related outcomes and do not report VTE or bleeding complications. Moreover, even when articles report risks of VTE and bleeding, key information is often missing or unclear.

Appropriate use of thromboprophylaxis requires assessment of the procedure-specific baseline risks for VTE and bleeding [13]. The absence of procedure-specific guidelines in general and gynecologic surgery is in considerable part due to the unavailability of credible baseline risk estimates. We will remedy this deficit by providing the first procedure and patient risk factor specific summaries for these fields. Our results will directly inform clinicians and guideline developers worldwide, resulting in rationalization of current practice and decrease in both under- and overuse of thromboprophylaxis, resulting finally in better patient outcomes and wiser use of limited resources [72].

We will communicate with key members of the societies (guideline organizations) and journals of general and gynecologic surgeries and present the results at major meetings. We will prepare “end of project packages” deliverable to these organizations and ensure that our dissemination strategy targets appropriate audiences. Where appropriate, our results will be packaged as actionable messages, including multiple versions from which the target audience can choose, “one sentence versions” highlighting implications, “one paragraph versions” reflecting importance, results, implications for decision-makers, and what should be done, full reports and decision aids. CLUE Working Group also has a website and YouTube channel, and we use Twitter for the dissemination of research findings.

## Supplementary Information

**Additional file 1.** PRISMA-P checklist.

## Data Availability

Not applicable.

## Abbreviations

ASH: American Society of Hematology
CLUE: Clinical Urology and Epidemiology
DVT: Deep vein thrombosis
EAU: European Association of Urology
ERAS: Enhanced Recovery After Surgery
GRADE: Grading of Recommendations Assessment, Development and Evaluation
PE: Pulmonary embolism
MeSH: Medical subject headings
PRISMA-P: Preferred Reporting Items for Systematic Review and Meta-Analysis Protocols
PROSPERO: International prospective register of systematic reviews
RCT: Randomized controlled trial
ROTBUS: Risk of Thrombosis and Bleeding in Urological Surgery
ROTBIGGS: Risk of Thrombosis and Bleeding in General and Gynecologic Surgery
VTE: Venous thromboembolism
